# Characterization of Different Commercial Dietary Supplements in the Peri-Weaning Period on Consumption and Growth Performance in C57Bl/6J Mice

**DOI:** 10.3390/ani10081284

**Published:** 2020-07-28

**Authors:** Angela M. Craig, Melanie L. Graham

**Affiliations:** 1 Research Animal Resources, University of Minnesota, Minneapolis, MN 55455, USA; 2 Veterinary Population Medicine Department, University of Minnesota, St. Paul, MN 55108, USA; graha066@umn.edu; 3 NAMSA, Minneapolis, MN 55443, USA; 4 Department of Surgery, University of Minnesota, St. Paul, MN 55108, USA

**Keywords:** mice, C57BL/6, nutrition, protein, pups, growth performance, refinement

## Abstract

**Simple Summary:**

The current study compares two different commercially available nutritional supplements promoted as caloric support for weanlings for acceptability and effect on weight and survival in mouse pups and lactating dams in the peri-weaning phase with standard diet mash or no supplementation. Our aim was to provide an independent characterization of nutritional supplementation on survival and weight gain in a commonly used mouse strain. These factors influence animal welfare and are essential for the success of a general breeding program. Similarly, supplementation in the peri-weaning phase can alter early nutrition depending on type, which can introduce unintentional confounding that affects the reliability of subsequent experimental outcomes. This study was also designed to consider pragmatic aspects including timing, cost-effectiveness, diet composition, and practicality in an effort to identify supplements capable of optimally supporting pup growth and survival for various research applications in diverse animal use programs.

**Abstract:**

This experiment was conducted to investigate the effects of common commercially available dietary supplementation in the peri-weaning period on feed intake, growth, and survival in C57Bl/6J mouse pups and lactating dams. A total of 96 pups and their dams were randomized to the control group or one of three nutritional supplement treatment groups: (i) control group without supplementation, or (ii) weanling-targeted Clear H_2_O gel (Gel), (iii) transgenic-targeted Bio-Serv dough (Dough), or (iv) dam diet as a mash (Chow), in the peri-weaning period (from 11 to 28 days). Stool was observed daily for a dye marker indicating supplement consumption. Pups were weaned at 21 days and followed for a total of 42 days. No pup morbidity or mortality was observed. There was a higher proportion of pups consuming dough and gel earlier than chow (*p* = 0.0091). The majority of treated pups (>95%) were consuming the supplement by day 23 (range 15–23), suggesting interplay between organoleptic properties of the supplement and pup maturity. All groups gained weight, with typical sexual dimorphism observed in the growth curves. Dough treatment led to significantly higher average daily gain in male pups (0.64 ± 0.03 g/d) as compared with controls (0.58 ± 0.03 g/d). The highest average daily gain in all groups was observed pre-weaning between days 21 and 28. Compared with controls, the weight gain slope was significantly higher in the Dough and Chow treatment groups and lower in Gel treatment groups, with a more pronounced effect in males. In this study, the composition of nutritional supplementation was the dominant factor in increasing the growth trend as opposed to energy density. Peri-weaning supplementation with Dough and Chow treatments improved pre- and post-growth performance in a comparable way and was more effective than Gel treatment during adaptation to solid feeding. Proper application of supplements to support weanlings can directly improve welfare and limit unintended experimental variability.

## 1. Introduction

Nutritional supplementation is a common practice for supporting laboratory mice that have special needs due to age, health status, phenotype, or experimental design [1,2,3]. Supplements can vary substantially in ingredient base, nutrient composition, and formulation as liquids, gels, doughs, whole foods, or pelleted or extruded diets. The cost, ease of delivery, palatability, sterility, advertised purpose, on-site experience, and perceived beneficial effects are some of the factors that influence choice of supplement. For these reasons, nutritional supplementation strategies in mice are largely empirical and vary even within a single institution.

Early nutrition is relevant from both the welfare perspective and use in experimental modeling, owing to the impact on growth, health, and disease susceptibility [4,5,6,7,8,9,10]. In laboratory mice, nutritional supplementation has been anecdotally promoted as a way to increase neonate weight gain, improve maternal success, reduce pup mortality through the weaning period, and support mice with developmental delays. This is especially relevant in the increasingly sought-after transgenic and knock-out mouse models where genetic changes may manifest as abnormal anatomy or behavior affecting feed intake. At the same time, dietary manipulation is not without consequences, and therefore may impact research results. Changes in postnatal development and growth long-term have been observed during intentional experimental manipulation [11,12,13,14,15].

There is limited data evaluating supplements promoted for use in the peri-weaning phase and no direct comparisons investigating which, if any, confer a clinically relevant benefit over the simplest approach of supplementing regular diet in accessible ways to pups [16,17,18,19].

The purpose of this study was to characterize supplement acceptability, time to utilization, effect on growth, and survival of pups using the C57Bl/6J mouse which is one of the most commonly used murine strains and background for many genetically modified lines [20]. We hypothesized that supplement composition and texture might influence appeal for consumption, rate of weight gain as a primary marker of growth, and survival. The present study compares three nutritional supplements, namely the weanling-targeted Clear H_2_O gel (Gel), transgenic-targeted Bio-Serv dough (Dough), and dam diet as a mash (Chow), with a control group without supplementation in the 11–28 day peri-weaning period and post-weaning performance.

## 2. Materials and Methods

All procedures in this study were approved by the Institutional Animal Care and Use Committee of the University of Minnesota, #1306-30738A.

### 2.1. Animals

Each dam gave birth to 5–10 pups and all dams’ parturition dates were within a two-day period. On postnatal (P) day P4 or P5, litters were either culled to a total of six pups (*n* = 13) or had a randomly selected pup cross-fostered into the litter to equalize the litter number to six (*n* = 3). Pups were toe-clipped for identification within the litter [21,22]. When possible, litters were comprised of even numbers of males and females. Of the 96 pups enrolled, 42 were male and 54 were female. To avoid bias in assignment, each of these litters with the dam was allocated to a study condition by drawing from a hat and assigning to the condition drawn. By chance, the supplement treatment groups were roughly equal in their sex distribution, while the control group contained twice the number of females than males. From P0 to P20, pups were housed with their dams. On day P21, pups were weaned and separated into same sex groups of littermates, and dams were singly housed. The study was concluded for all mice on day P42. The dams were verified free of all common murine pathogens including EDIM, Mycoplasma pulmonis, MHV, MNV, MPV, MVM, TMEV, and Sendai. Parasite testing for *Myobia musculi, Radfordia affinis, Radfordia ensifera, Mycoptes musculinus*, and all species of known pinworms was negative. The room was also monitored using bedding contact sentinel mice and no outbreaks of disease or parasites were detected one year prior to, during, or one year after the study.

### 2.2. Husbandry

The dams and litters were housed in autoclaved static microisolator cages (Allentown Incorporated, Allentown, NJ, USA) with irradiated corn cob bedding (7902, Harlan, Madison, WI, USA), irradiated extruded diet (Teklad Global 2919, Harlan, Madison, WI, USA), and autoclaved municipal tap water. Housing and husbandry were provided in a barrier rodent facility within an AAALAC-accredited unit, and met expectations set in the Guide for the Care and Use of Laboratory Animals [23].

### 2.3. Dietary Supplementation

The dams and their litters were randomized to one of four groups: dough nutritional supplement (Dough), gel nutritional supplement (Gel), moistened regular diet nutritional supplement (Chow), or control, and remained together until weaning (P21).

The dam diet in the feed hopper was available ad lib continuously. Treated groups were additionally provided either Dough (Transgenic Dough Diet, Bio-Serv, Frenchtown, NJ, USA), Gel (DietGel Boost, ClearH20, Portland, ME, USA), or Chow (irradiated extruded diet, Teklad Global 2919, Harlan, Madison, WI, USA provided as a mash). A comparison of the nutritional composition of the products is summarized in Table 1 [24,25,26]. The supplements were offered from day P11 to P28 only.

The supplements were prepared fresh daily within 30 min of placement in the cage and provided in a petri dish (USA Scientific, Ocala, FL, USA) on the cage floor. In the Dough and Gel groups, the supplements were hand-packed to a level even with the top of the dish lid. The lid volume was 17.24 cm^3^. In the Chow group, three complete diet pieces were placed in each dish and weighed at dry weight, then soaked in water for 30 min to soften to a mash consistency and re-weighed to estimate moisture content. The control dishes were left empty. A tasteless, non-toxic, human-grade food dye (Royal Blue Icing Color, Wilton, Woodridge, IL, USA) was painted liberally over the gel, dough, and moistened extruded diet. When each day’s supplement was provided, the previous day’s Petri dish was removed. The amounts of fresh supplement provided were calculated to offer an ad libitum consumption opportunity.

### 2.4. Data Collection and Animal Handling

The pups and dams were weighed on postnatal days 11, 13, 15, 17, 19, 21, 23, 25, 27, 28, 31, 35, 38, and 41. Weights were measured and recorded to the nearest hundredth of a gram. The scale (EJ-120, A&D Company, San Jose, CA, USA) was calibrated before weighing commenced and was checked with a standard weight for accuracy between each litter of pups.

**Figure 1:**
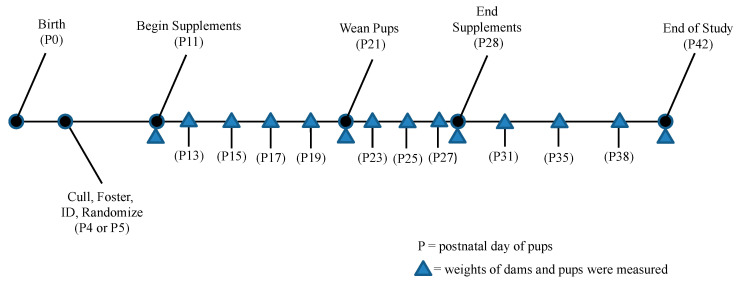
Experimental timeline as a function of pup age in days. Postnatal days indicated by “P” and shaded triangles indicate weight measurement in relation to experimental supplementation.

During the supplementation period, the pups and dams were gently restrained by hand and examined daily for the presence of dye in their stool to confirm whether the supplement had been consumed by the individual. Confirmation of consumption was recorded on the first day of observed dyed feces or dye staining on the anus. In the test groups, the stool was a normal tan to brown color prior to supplementation and appeared green to blue in color when the supplement was consumed. The control group animals were handled in the same way and had a normal tan to brown stool color throughout the study. The experimental timeline is summarized in Figure 1.

### 2.5. Statistics

Demographic data are expressed as mean ± standard error (SE) in multivariate analysis. Statistical analyses were performed using the GraphPad 6 Prism program (GraphPad Prism v6, GraphPad Software Inc., La Jolla, CA, USA) and one- and two-way analysis of variance (ANOVA) tables were generated using the open source tool designed by Assaad et al. [27,28]. Time to event (supplement use) curves for each group of mice were estimated by using the Kaplan–Meier method, compared by using the log-rank test, and expressed also as median (minimum–maximum). The primary outcome measure was differences in bodyweight change from baseline (P11) between supplemented groups and the control group, normality was assessed using the Shapiro–Wilk test and variance homogeneity by Bartlett’s test, and linear regression included subject-specific intercept and slope terms. Two-way ANOVA with Tukey was performed to evaluate the relationship between supplement, sex, and together evaluated for interaction. Average daily gain measurements in the four groups were compared using a one-way Brown–Forsyth ANOVA, adjusting the F ratio and degrees of freedom to adjust for heterogeneity of group variances with Tukey or Dunnett corrections for multiple comparisons as indicated in figures/tables, and stratified by sex. Normally distributed data were additionally analyzed using multiple comparisons on pairs of groups using Bonferroni correction. *p*-values presented are based on two-tailed tests, and values < 0.05 were regarded as significant.

## 3. Results

### 3.1. Survival and Birth Weights

**Figure 2:**
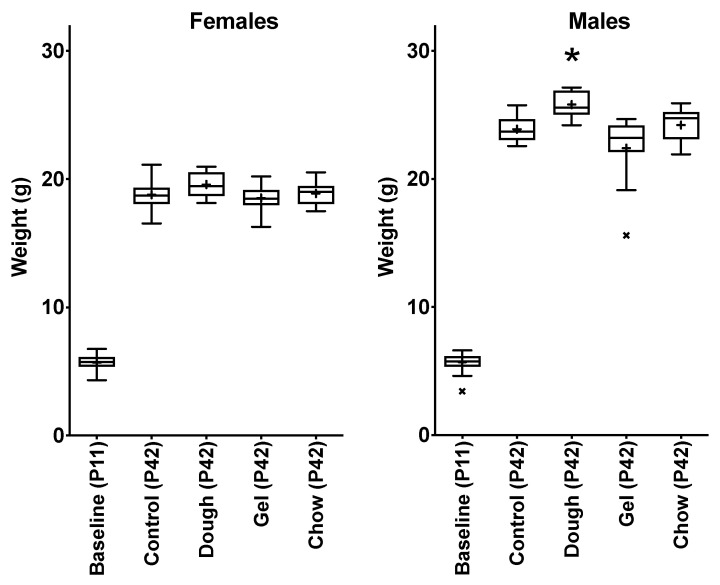
Weight distribution at baseline and endpoint by sex. At baseline, the cohorts were normally distributed. Two weeks after the withdrawal of supplement, weights were normally distributed except for the Gel-supplemented male cohort (*p* = 0.0476) and the average weights of males that were supplemented with Dough were significantly higher than controls (*p* = 0.0016), indicated with an asterisk.

**Figure 3:**
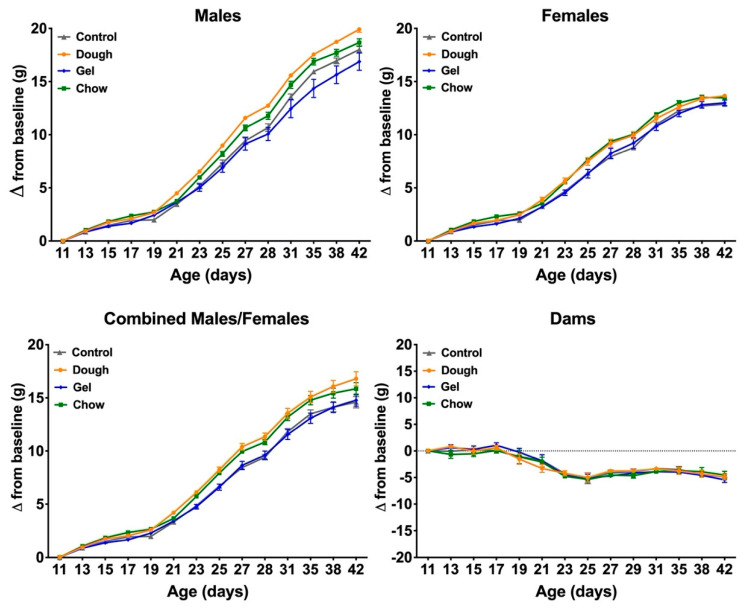
Body weight comparison with control by treatment group. Symbols indicate the average weight from baseline (P11) by postnatal day and error bars indicate SEM. Dough (orange), Gel (blue), and Chow (green) as compared with controls (grey) and stratified by sex. Assessment of growth trends revealed the Dough slope (0.63 ± 0.01, intercept = 2.83, R^2^ = 0.88, *p* < 0.0001) and Chow slope (0.60 ± 0.01, intercept = 2.89, R^2^ = 0.90, *p* = 0.0095) were significantly higher than the control group slope (0.55 ± 0.01, intercept = 1.81, R^2^ = 0.90), while Gel was not significant. When the growth trend was stratified by sex, males consuming the Dough supplement had a significantly higher slope (0.74 ± 0.01, intercept = 4.8, R^2^ = 0.96, *p* = 0.0027) and males consuming the Gel supplement had a significantly lower slope (0.61 ± 0.02, intercept = 4.0, R^2^ = 0.87, *p* = 0.0331) when compared with male controls (0.67 ± 0.02, intercept = 4.5, R^2^ = 0.95), and Chow was not significant.

All sixteen dams and 96 pups enrolled in the study survived the entire data collection period from birth/parturition through day P42. At enrollment, one pup was smaller than average (Figure 2), and this animal’s litter had been randomized to the gel-supplemented group. This animal was not a member of the three litters which received a foster pup to make a total of six neonates. Despite starting the study at a lower weight than the cohort, this animal gained weight, and had a normal growth trajectory (Figure 3), and at the endpoint all animals were within a normal distribution except a single male in the Gel group with attenuated gain comparative to other male pups in the group (Figure 2).

### 3.2. Time to Consumption for Pups

**Figure 4:**
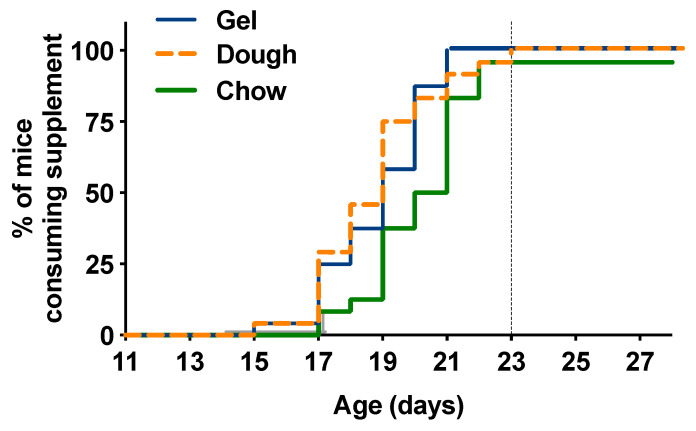
Kaplan–Meier time to event (supplement utilization) curve, by supplement group. Dough and Gel supplements were consumed earlier than Chow (*p* = 0.0091). The majority of animals (>95%) were consuming the supplement by day 23, indicated by the dotted line.

Gastric transit time in mice varies depending on the composition of the diet consumed, the strain of mouse, the population of gut microbial flora, and the measurement technique (e.g., observation of markers via imaging, chemical labels, or indigestible dye marker) [29,30,31,32]. The total transit gastrointestinal time reported in mice is between 6–8 h [31], so for the purpose of this study, time to consumption was recorded as the day in which dyed feces were observed in each supplemented mouse. Different types of supplements did not alter overall consumption, and practically all pups consumed the supplement by day 23 regardless of type. The proportion of pups consuming the supplement was 100% for Dough, 100% for Gel, and 96% for Chow. The median (minimum–maximum) time (days) for pups to start the test supplements was 19 (15–23) for Dough, 19 (15–21) for Gel, and 20.5 (17–22) for Chow (Figure 4). Log-rank test results for between-group differences in time to supplement use were calculated, and dough and gel supplements were consumed earlier in a higher proportion of animals when compared with moist chow supplements (*p* = 0.0091).

### 3.3. Time to Consumption and Weight of Dams

All dams in treated groups consumed the supplement and had dyed feces by post-partum day 12. No effect on weight was observed despite the differences in formulation and nutritional composition of the supplements. There was no statistical difference between the supplemented dams’ weight trends or in comparison to the control dams’ weights during lactation or involution.

### 3.4. Comparison of Growth between Pup Groups

The postnatal days evaluated during this study can be logically divided into phases related to pup development. The period from P11–P21 (2–3 weeks of age) is the pre-weaning phase during which the pup has maternal care and access to dam’s milk. In the early post-weaning phase from P22 to P28 (4 weeks of age), the pup has been separated from its dam and must develop fully precocious behavior. Taken together, these can be considered the peri-weaning phase. During the late post-weaning period of P29–P42 (5–6 weeks of age), the pup continues to grow and develop towards sexual maturity and adulthood.

Two-way ANOVA (Table 2) confirmed the observation in the growth curves that Dough and Chow supplementation resulted in higher weights as compared with controls post-weaning. Similarly, the weights in males are higher than in females, supporting the main effect of sex on growth. There was no significant interaction between main effects, supplement, and sex, across groups at baseline or end of each development phase (Table 2), however there was an evident trend towards an interaction between diet and sex in supplemented males in the Dough and Chow groups.

Growth curves by supplement, which were stratified by sex, are presented in Figure 3. The expected sexual dimorphism in growth rates is evident, where around P28 in all conditions, males were already significantly heavier than females (*p* < 0.05). Rate of growth was calculated as slope of the regression line relating body weight over time. One-way ANOVA comparing the slopes of the control group with Dough and Chow revealed significant differences *p* < 0.0001 and *p* = 0.0095, respectively, while Gel was not significant. When stratified by sex, similarities in slopes were observed between female controls, Dough, Chow, and Gel. However, significant differences (*p* < 0.0001, F = 12.82) were observed in slopes between treatments in males. One-way ANOVA comparing the slopes of male controls with Dough and Gel revealed significant differences *p* = 0.0027 and *p* = 0.0331, respectively, but not Chow.

Pup weight gain was similar across groups in the early postnatal period. Beginning on P19, the Dough growth curves had a more pronounced upward trend (Table 2, Figure 3), followed by the Chow curve beginning on P25. The average daily gain (ADG) during this period was in general agreement with the slope (Table 3). Differences between supplements and effect on gain were observed between groups depending on pup development phase, and were prominent during the supplementation period of P11 to P28 when analyzed by one-way ANOVA.

Of the three supplements, the overall growth rate was the highest in the Dough pups as compared with controls, with a stronger effect in males. Consistent gain in Dough and Chow as compared with controls was observed around the time of weaning, especially in Dough-supplemented males.

There was no apparent statistically or clinically relevant benefit in using Gel as compared to controls. The Gel group did not demonstrate a measurable weight gain benefit compared to controls. Moreover, the growth rate was lower in both females and males as compared with controls (Figure 3). The growth rate was slower, and weights were significantly (*p* = 0.033) lower in males consuming Gel versus females and in comparison with controls.

On P42, the average body weight was comparable between groups in females. In males, the highest body weight was observed in the Dough group, which was significant when compared with controls (*p* = 0.0016, Figure 2).

## 4. Discussion

Early feeding behavior in mice has been incompletely characterized, leading to uncertainty regarding the utility and timing of supplements. Although there are commercial supplements targeting improved weanling performance, an independent assessment of supplement performance in mice, even a common strain, was lacking. The purpose of this study was to examine the value of three different commercially available diet supplements pre-and post-weaning in C57Bl/6J mouse pups to determine if supplementation confers a survival or growth advantage. Weaning is a challenging phase related to rapid growth and development together with the shift to a diversified diet. This study presents novel observations of pup feeding behavior during this period and factors that might influence time to consume supplementary feed and how composition impacts the rate of growth, morbidity, and survival. Characterizing the effect of nutritional supplementation during this period of growth and development can impact husbandry and veterinary care decisions at the vivarium or institutional level, and has the potential to promote improved outcomes for mice with known reproductive difficulties related to pup survival and maturation. While pups acquire most of their energy from consumption of maternal milk, supplements may play an important role in buffering limited milk yields in large litters or late lactation, adaptation to solid food after weaning, and support for animals with genetic alterations that affect nutrition. Supplements were offered starting on day 11 and continued only through day 28 to model the common practice in breeding colonies of defined periods of supplementation for weaned pups.

### 4.1. Supplement Nutritional Profile and Influence on Time to Consumption

Commercial supplements Gel and Dough were selected based on claims of ideal support for “weanlings” and compared with standard Chow diet formulated to support growth. The comparison of the guaranteed analyses (Table 1) shows that Dough and Chow supplements were more similar in composition, and reflected the typical carbohydrate shifted diet fed to mice with ≥15% protein [33]. The Gel supplement was highest in fat and lowest in protein. As fed, Dough had the highest energy density followed by Gel and then Chow. Beyond nutrient content, there are other differences between supplements, including ingredient base and physical form. Mice will overconsume high-fat diets or diets high in simple sugars [34]. The Dough’s top ingredient is the simple sugar sucrose. The Gel’s top ingredient is peanut butter, which is high in fat, followed by molasses, a simple sugar. The dough had a thick paste consistency with a bacon scent and the Gel had a nut butter consistency and peanut scent. We hypothesized that these organoleptic properties of the supplement might impact the order of consumption. Tooth eruption in C57Bl/6J mice occurs at approximately 11 days of life. The earliest evidence of supplement consumption by dyed feces did not occur until day P15, so preference for texture did not appear to be associated with the development of teeth. Approximately 50% of pups were reliably consuming supplements by day 19, and >95% by day 23. The start of consumption instead appeared to be linked to the pups’ maturity and concurrent decrease in neophobia [35]. Supplements designed with an appealing texture and odor, Dough and Gel, had a faster time to consumption (Figure 4). The moist extruded regular diet was last in order of consumption. These flavors, also present in maternal milk, may have influenced interest in the supplement [35,36].

The earliest period of consumption of dietary supplements is not only interesting for pups which benefit from maternal care, but also for orphaned pups where it could have even greater importance. The time to consumption data in this study suggest that orphaned pups might not be able to take advantage of supplementation on their own until day P14 (the day prior to the earliest evidence of dyed feces) where ambulation is necessary to reach the diet. Further studies could be initiated to understand how supplemental diets may be introduced at pre-weaning time points to serve as primary nutrition for pups without access to dam’s milk and maternal care, and where fostering is not an option. The influence on weight gain and survival in this stressful deprivation condition could be substantial.

### 4.2. Survival

In this study, there was no mortality, and pups in this study exceeded typical survival of C57Bl/6J pups where mortality is typically about 13% as reported by a commercial breeder [37]. Pup mortality during the pre- and post-weaning periods includes factors related to behavior, nutrition, litter size, and disease. Dams tested negative for murine diseases and parasites, and disease was not a factor growth or survival. Nutritionally related deaths in the pre-weaning period are attributed to an inadequate supply of dam’s milk and in the post-weaning period, small size or weakness can limit access to the feed hopper and water source. Since the survival rate was equivalent in control and Chow groups, dam supplementation utilization likely had little or no effect. Litter size is another factor that influences both growth and overall survival [38]. The moderate pup burden on the dam, due to intentional experimental culling of litters to six pups, is likely to have positively impacted early growth and survival since available milk was shared among fewer pups.

### 4.3. Comparison of Weight Gain

In the early pre-weaning period, all supplements performed relatively equally and no statistically significant weight gain in any group was evident before P19. Mouse pups exhibit introductory feeding behavior similar to creep feeding in other species [39,40], and regardless of the supplement consumption pattern in the pre-weaning period, the dam’s milk remains the primary source of nutrition for pups at this age. The experiment was not designed to quantify the exact amounts of each available food item consumed by individual pups, including dyed supplement, dam’s milk, and crumbs or dust in the bedding below the regular diet in the feed hopper. While this may have impacted individual pup data, all pups had access to these same items equally, blunting the impact of these differences when all pups were evaluated together.

Providing supplement could prevent dams from losing excessive body reserves during lactation by reducing pup milk demand. However, none of the three supplements showed differences among the healthy, lactating adult female mice. Unless there are known dam-related nutritional concerns which would negatively impact pre-weaning pups, in terms of cost-benefit for pups and dams, it does not appear advantageous to provide supplementation before pups are weaned beyond the potentially influencing time to supplement consumption for pups. This may be relevant for institutions or experimental designs that require early weaning.

The enhanced weight gain in the Dough treatment groups, particularly males, may have been related to palatability or the higher nutritional density (Table 2). The greatest gains were during the post-weaning period when there was no access to the dam but still access to the supplement (Table 3). Supplement consumption was significantly associated with higher post-weaning average daily gain as compared with controls, except for Gel-treated males. The Gel’s energy density was between the Dough and Chow, but lower in protein and higher in fat composition compared with the other supplements. Mice will overconsume high-fat diets or diets high in simple sugars [34]. The Dough’s top ingredient is the simple sugar sucrose. The Gel’s top ingredient is peanut butter which is high in fat followed by molasses, a simple sugar. The low ADG in the Gel-treated groups is likely related to the preferential consumption of the supplement which is low in protein, since protein restriction is known to slow growth rates in mice [41]. This finding is consistent with previous reports utilizing this gel where decreased survival rates and lower average birth weights were observed with the conclusion that this gel was probably not suitable for use with breeders and pups [19]. The gel had no deleterious effects on the weight of dams in our study. For growing animals, the decision to provide gel formulations, especially those low in protein, should be weighed against the goals for supplementation. For a combination of hydration and nutrition in weak adult animals, gels may be useful, but as a neonate or weanling supplement, they appear less advantageous.

These data suggest that supplementation with the Dough in the early post-weaning period (P21–P28) will increase weight the most within the P21–P42 timeframe for pups with maternal care through weaning. Interestingly, the ADG achieved with Chow, that was simply a mash of balanced standard mouse diet, was almost equivalent to Dough supplements. Given this observation, easy access to the feed source for newly weaned pups confers an advantage even without uniquely formulated commercial supplements. In the potentially stressful period of the early post-weaning phase, this simple refinement in presentation ensures easy access to nutrition. Facilities that routinely provide standard diet on the cage floor to weanlings may experience similar pup support because of improved accessibility, however experimental evaluation is needed for confirmation since differences in pellet texture/hardness have been previously shown to affect growth [42]. There may be cost and convenience advantages to using Chow over Dough, but also a scientific preference for Chow since mice are not exposed to higher levels of fat and simple sugars early in life that may lead to persistent behavioral change or metabolic alteration [41,43,44,45,46,47]. The persistent increased weight in male pups treated with Dough as compared with controls (*p* = 0.0476) suggests there is impact of this supplementation beyond the intended peri-weaning period. Husbandry practices are not benign and early interventions, when not applied equally, may introduce unintentional variability. A limitation of the study was that it was not designed to evaluate the effects of continuing supplementation past P28 and metabolic or immunologic alterations were outside the scope of this evaluation.

The use of supplements is similarly dependent on clinical relevance and feasibility. There are costs related to the purchase of the products and the time it takes a technician to add them to the cages. Pre-administration storage is also a consideration. The Chow was easy to provide since it was a readily available diet and simple to make into a mash. Additionally, it required no special storage and introduced no new nutritional variables versus the control. Factoring in only the cost of the supplements, and not the technician time to administer them, the Dough is a more expensive option compared with Chow and must be refrigerated until use. However, the added product cost could be outweighed by improved pup survival and weight gain in strains with breeding difficulties. When commercial supplements are not scientifically appropriate, financially feasible, or practical to administer, a mash of the regular diet is a highly useful alternative with equivalent success in increasing ADG with the added benefit of using the diet the mouse will eventually consume in adulthood to limit confounding based on diet exposure.

## 5. Conclusions

This preliminary study provides a starting point for developing strategic, cost-effective, and data-driven pup supplementation by highlighting how the use and timing of commercial supplements promoted weanlings’ transition to a diversified diet, growth, and survival in a commonly used strain of mouse that is frequently also used as a background for genetically modified mice. This study did not explore the mechanisms underlying these differences that may have profound effects on subsequent scientific use. A plan for weanling supplementation at a vivarium or institutional level can enhance rigor in husbandry practices by unifying the practices for supporting strains with slow pup maturation or poor survival. Likewise, the characterization of these specific effects of supplementation exposes where they may be incompatible with future scientific use and should be reconsidered or be probed more deeply for effects beyond the primary goals of growth and survival. This study demonstrates that purposeful supplementation can serve as an important refinement to improve welfare and breeding colony management.

## Figures and Tables

**Table 1:** Comparison of guaranteed analysis “as fed” reported by the manufacturer.

	Manufacturer (Product ID)	Protein %	Fat %	Carbohydrate %	Moisture %	Energy Density ^a^ Kcal/gram
**Dough**	Bio-Serv (Transgenic Dough Diet)	21.2	12.4	46.5	<12	3.83
**Gel**	ClearH_2_O (DietGel Boost)	9.9	21.6	37.8	25–30	3.69
**Chow** ^b^	Teklad (2919)	19.0	9.0	44.9	47–59 ^c^	3.3

^a^ Energy density is a calculated estimate of metabolizable energy based on the Atwater factors assigning 4 kcal/g to protein, 9 kcal/g to fat, and 4 kcal/g to available carbohydrate. ^b^ The extruded diet pellet reported by manufacturer for guaranteed analysis basis. ^c^ Estimated moisture content following mash preparation.

**Table 2:** Change in weight with dietary supplementation and/or sex.

Phase	Control		Dough (DNS)		*p-*Value		
	F (*n* = 16)	M (*n* = 8)	F (*n* = 12)	M (*n* = 12)	Diet	Sex	D◊S ^1^
P11	5.9 ± 0.1	5.9 ± 0.2	5.9 ± 0.2	5.9 ± 0.1	0.95	0.86	0.92
P21	9.2 ± 0.2 ^b^	9.3 ± 0.3 ^a,b^	9.8 ± 0.4 ^a,b^	10.4 ± 0.2 ^a^	0.001	0.14	0.44
P28	14.7 ± 0.3 ^c^	16.5 ± 0.5 ^b^	15.9 ± 0.5 ^b,c^	18.6 ± 0.2 ^a^	<0.001	<0.001	0.20
P42	18.8 ± 0.3 ^c^	23.9 ± 0.4 ^b^	19.6 ± 0.3 ^c^	25.8 ± 0.3 ^a^	<0.001	<0.001	0.07
Phase	**Control**		**Moist (MNS)**		***p*-Value**		
	**F** **(*n* = 16)**	**M** **(*n* = 8)**	**F** **(*n* = 13)**	**M** **(*n* = 11)**	**Diet**	**Sex**	**D◊S ^1^**
P11	5.9 ± 0.1 ^a^	5.9 ± 0.2 ^a,b^	5.4 ± 0.1 ^b^	5.5 ± 0.1 ^a,b^	0.002	0.73	0.53
P21	9.2 ± 0.2	9.3 ± 0.3	9.0 ± 0.2	9.3 ± 0.2	0.63	0.23	0.71
P28	14.7 ± 0.3 ^c^	16.5 ± 0.5 ^a,b^	15.5 ± 0.2 ^b,c^	17.3 ± 0.4 ^a^	0.003	<0.001	0.98
P42	18.8 ± 0.3 ^b^	23.9 ± 0.4 ^a^	18.9 ± 0.3 ^b^	24.2 ± 0.4 ^a^	0.012	<0.001	0.72
Phase	**Control**		**Gel (GNS)**		***p*-Value**		
	**F** **(*n* = 16)**	**M** **(*n* = 8)**	**F** **(*n* = 13)**	**M** **(*n* = 11)**	**Diet**	**Sex**	**D◊S ^1^**
P11	5.9 ± 0.1	5.9 ± 0.2	5.5 ± 0.2	5.5 ± 0.3	0.07	0.926	0.92
P21	9.2 ± 0.2	9.3 ± 0.3	8.7 ± 0.4	9.2 ± 0.4	0.34	0.332	0.68
P28	14.7 ± 0.3	16.5 ± 0.5	14.7 ± 0.6	15.6 ± 0.8	0.73	0.018	0.37
P42	18.8 ± 0.3 ^b^	23.9 ± 0.4 ^a^	18.5 ± 0.3 ^b^	22.4 ± 0.8 ^a^	0.68	<0.001	0.21

Two-way ANOVA of the effects of supplement (control, Dough, Chow, Gel) and sex combinations on weight. Values are means ± SEM. ^a–c^ Means in a row without a common superscript letter differ (*p* < 0.05) as analyzed by two-way ANOVA and the TUKEY test. ^1^ D ◊ S = Diet ◊ Sex interaction effect.

**Table 3:** Average daily gain (ADG) with dietary supplementation by growth phase in males and females.

Females	Control (*n* = 16)	Gel (*n* = 13)	Dough (*n* = 12)	Chow (*n* = 13)
ADG 11–21	0.32 ± 0.01 ^b^	0.32 ± 0.01 ^b^	0.39 ± 0.02 ^a^	0.36 ± 0.01 ^a,b^
ADG 22–28	0.79 ± 0.02 ^a,b^	0.86 ± 0.06 ^a,b^	0.87 ± 0.03 ^a,b^	0.93 ± 0.02 ^a^
ADG-29–42	0.29 ± 0.01	0.27 ± 0.01	0.27 ± 0.03	0.24 ± 0.01
Cumulative ADG 11–42	0.42 ± 0.01	0.42 ± 0.01	0.47 ± 0.06	0.43 ± 0.01
**Males**	**Control (*n* = 8)**	**Gel (*n* = 11)**	**Dough (*n* = 12)**	**Chow (*n* = 11)**
ADG 11–21	0.35 ± 0.02 ^b^	0.36 ± 0.02 ^b^	0.45 ± 0.01 ^a^	0.38 ± 0.01 ^b^
ADG 22–28	1.03 ± 0.03 ^a,b^	0.92 ± 0.08 ^b^	1.18 ± 0.01 ^a^	1.15 ± 0.04 ^a^
ADG-29–42	0.52 ± 0.02	0.49 ± 0.03	0.51 ± 0.01	0.49 ± 0.01
Cumulative ADG 11–42	0.58 ± 0.01 ^a,b^	0.54 ± 0.03 ^b^	0.64 ± 0.01 ^a^	0.6 ± 0.01 ^a,b^

One-way ANOVA of the effect of supplement (control, Dough, Chow, Gel) stratified by sex. Values are means ± SEM. ^a,b^ Means in a row without a common superscript letter differ (*p* < 0.05) as analyzed by one-way and the TUKEY test.

## Abbreviations

P	postnatal day
EDIM	epizootic diarrhea of infant mice
MHV	mouse hepatitis virus
MNV	mouse norovirus
MPV	mouse parvovirus
MVM	minute virus of mice
TMEV	Theiler’s encephalomyelitis virus
